# Optimization design of internal space layout of three-bedroom residential apartment based on IGA and DE algorithm

**DOI:** 10.1371/journal.pone.0326153

**Published:** 2025-07-07

**Authors:** Ling Zhao, Baijun Li

**Affiliations:** 1 School Design, Nanjing University of the Arts, Nanjing, China; 2 School of Design and Innovation, Shenzhen Technology University, Shenzhen, China; 3 Overseas Chinese Town Holdings Company (OCT Group), Shenzhen, China; The Hong Kong Polytechnic University, HONG KONG

## Abstract

To solve the problems of insufficient global optimization ability and easy loss of population diversity in building interior layout design, this study proposes a novel layout optimization model integrating interactive genetic algorithm and improved differential evolutionary algorithm to improve the global optimization ability and maintain population diversity in building layout design. The model characterizes room functions and spatial locations through binary coding, and uses dynamic fitness function and backtracking strategy to improve space utilization and functional fitness. In the experiments, optimization metrics such as kinematic optimization rate (calculated based on the shortest path and connectivity between functional areas), space utilization rate (calculated by the ratio of room area to total usable space), and functional fitness (based on the weighted sum of users’ subjective evaluations and functional matches) all perform well. Quantitatively, it is found that the model achieves 94.76% in terms of motion optimization rate, the highest space utilization rate is 96.6%, functional fitness is 9.4, and user satisfaction is close to 94.21%. The optimization results show that the proposed method has significant advantages in improving space utilization and meeting personalized design needs. However, despite the good optimization results, the method still faces the problem of improving the optimization ability under high-dimensional space and complex constraints. This study provides an efficient solution for intelligent building layout design and has certain practical value.

## 1. Introduction

The layout of internal spaces in buildings is an important component of architectural design, which has a direct influence on the realization of building functions, the improvement of usage efficiency, and the quality of life and experience of users. With the complexity of residential functional requirements and the improvement of personalized requirements, the traditional method of designing the internal space layout (ISL) of buildings has been difficult to meet the needs of modern architectural design. As a comprehensive research method, ISL optimization has gradually received extensive attention from both academia and practice [1,2]. However, the existing optimization methods mostly rely on standardized models, ignoring the personalized design requirements and the subjective experience of space users. Especially under diversity maintenance, global optimization capability and complex constraints, the existing methods are still insufficient. Therefore, how to take into account the personalization of the space and the user’s needs on the basis of efficient optimization has become an important topic in current research. Lu Y et al. proposed an optimization method that combines parametric design with Generative Adversarial Networks (GANs). Experimental results showed that the method could significantly reduce the computational cost and provide architects with a fast design strategy [3]. However, the method lacks the consideration of users’ subjective experience and fails to effectively support personalized design, especially in the case of meeting different users’ needs. Skrzypczak I et al. used laser 3D scanning technology to improve the measurement accuracy of interior design, and combined building information modeling and geospatial environments to generate more accurate interior layout schemes [4]. Nevertheless, this method relied on single building data and could not fully consider the functional requirements and user experience in spatial layout. Li S et al. proposed a spatial layout model combining Genetic Algorithm (GA) and Differential Evolutionary (DE) algorithm, and the optimization results showed that this method had a significant improvement in space utilization [5]. However, this optimization method neglected the flexible adjustment of personalized needs and the algorithm’s local optimization ability was still limited when facing complex real-world scenarios. The deep learning algorithm for layout design based on adversarial neural networks proposed by Sun Y has made progress in improving the subjective evaluation [6]. However, it had high computational requirements and lacked the in-depth integration of personalized needs of spatial users. Yu F et al. introduced the an interactive DE algorithm based on backtracking operator to improve the execution efficiency and convergence speed, but this method still could not effectively maintain the diversity in the layout optimization process [7].

Intelligent optimization algorithms are algorithms that automatically search for optimal solutions by mimicking natural evolution or other intelligent behaviors, often combining global search capabilities and local optimization strategies to solve complex optimization problems. In intelligent optimization algorithms, GA is widely used for optimizing ISL in buildings due to its flexibility and global optimization capabilities. DE, as a classic evolutionary algorithm, also performs well in global optimization problems due to its simplicity, efficiency, and fast convergence speed [8,9]. Harshalatha et al. optimized the energy performance of the building through GA. The experimental results showed that the method performed well in terms of spatial configuration and energy saving, but it lacked the integration of personalized design of indoor space layout with user feedback [10]. Li Y et al. proposed a multidimensional evaluation layout system based on the health needs of the elderly, and optimized the spatial layout of the urban plaza, to balance the needs of urban development with the human-centered design concept [11]. Although the method is effective in specific populations, it is still difficult to adapt to diversified user needs in universal residential or office space layout. Fakhr B V et al. proposed a Pareto frontier optimization method to optimize the thermal lighting comfort and energy saving of indoor layouts, but the method failed to consider in depth the effects of different cultures and individualized needs on spatial design [12]. Huang P Q et al. combined DE algorithm to develop a layout assistance framework for wireless communication systems to optimize the layout of communication systems, but the method did not address the human behavioral and cognitive factors in spatial design [13]. AlSaggaf A et al. proposed a methodology for integrating building information modeling and Geographic Information System (GIS) models for site layout planning. The method still lacks a cultural adaptation analysis although it has demonstrated excellent usability in several real-life cases [14]. Min X et al. proposed a museum gallery graphic design method based on Conditional Generative Adversarial Network (CGAN), which can help architects work more effectively. The results showed that the method could generate museum gallery floor plans with a certain regularity based on given conditions rather than purely random generation [15]. Lin H et al. utilized the technical advantages of CGAN in image generation and style transformation to create a method for independently designing a specific facade decorative style. The results showed that the method could better identify and generate decorative styles for historic neighborhoods than traditional methods. It could realize the whole or partial scheme design of the facade [16]. The visualization analysis of the above literature is shown in Table 1.

**Table 1:** Comparison of differences in literature methods.

Method	Advantages	Disadvantages	Reference number
Optimization method combining parametric design and GANs	Significantly reduces computational cost, providing a quick design strategy	Lacks consideration of user subjective experience, does not effectively support personalized design	[3]
Laser 3D scanning technology combined with building information modeling	Improves measurement accuracy of indoor design, generating more accurate layouts	Relies on single building data, cannot fully consider functional needs and user experience	[4]
Space layout model combining GA and DE	Significant improvement in space utilization rate	Ignores personalized needs, with limited local optimization capability	[5]
Deep learning algorithm for layout design based on GANs	Improves subjective evaluation and adaptability	High computational demand, lacks deep integration of personalized needs	[6]
Interactive DE algorithm with backtracking operator	Improves execution efficiency and convergence speed	Cannot effectively maintain diversity in the layout optimization process	[7]
GA optimizing building energy performance	Optimizes building energy performance, improves space configuration and energy saving	Lacks integration of personalized design and user feedback	[10]
Multi-dimensional evaluation layout system based on elderly health needs	Balances urban development and human-centered design, focusing on elderly health	Difficult to adapt to diverse user needs, primarily focused on specific groups	[11]
Pareto frontier optimization method for indoor layout of thermal lighting comfort and energy savings	Optimizes thermal lighting comfort and energy savings	Does not fully consider cultural and personalized needs	[12]
Wireless communication system layout support framework combined with DE algorithm	Optimizes communication system layout, improving efficiency	Does not address human behavior and cognitive factors in spatial design	[13]
Site layout planning method integrating BIM and GIS models	Provides 4D visualization and dynamic conflict detection, improving usability	Lacks cultural adaptability analysis	[14]
CGAN-based graphic design method for museum exhibition halls	Generating regular museum gallery plan results	Not very scalable	[15]
CGAN-based decorative styles for neighborhood facades	Higher validity and better generation	Lack of timeliness	[16]

In summary, existing research has made significant progress in improving the efficiency and accuracy of architectural ISL design, but there are still some gaps in these methods as follows: ① Lack of personalized design: most of the existing optimization methods rely on standardized layouts, failing to fully consider the user’s subjective needs and emotional responses. Despite some progress in space utilization and functional matching, the adaptability of these methods to different users is poor. Although some progress has been made in space utilization and functional matching, the adaptability to different users’ habits, psychological needs and cultural backgrounds is poor. ② Problem of local optimum and global optimization: although traditional GA and DE algorithms have strong global search capability, they are easy to fall into local optimum under complex constraints and diversity demands, and lack effective diversity maintenance mechanism, which leads to unsatisfactory optimization results. ③ Neglecting cognitive and behavioral factors: many existing methods focus on functional optimization of space, but fail to combine cognitive psychology and environmental behavior, ignoring the impact of layout on human comfort, stress, attention, and overall health. Meanwhile, most current internal space optimization studies emphasize computational modeling and algorithmic implementation, but lack professional parameter descriptions in architectural design practice, such as opening control, flow organization, and furniture scale coordination. This discrepancy limits the implementation of AI models in the actual architectural design process. To enhance the connectivity between this study and the architectural profession, this study introduced spatial organization principles, such as the ratio of openings and depths, and the guidance of human movement and sight lines, into the model design phase to ensure that the optimization results have implementability and architectural consistency.

In addition, GA is prone to local optimization and has low search efficiency under complex constraints, while DE algorithms are deficient in population diversity maintenance. To overcome the limitations of a single algorithm and address the above gaps, the study proposes an optimization model based on a hybrid of Interactive Genetic Algorithm (IGA) and DE. By combining the user-subjective feedback mechanism of IGA with the global search capability of DE, the hybrid method can improve the adaptability and optimization effect of personalized design while ensuring global optimization. Compared with other algorithms such as particle swarm optimization or artificial bee colony algorithm, the combination of GA and DE can better balance the global search and local optimization capabilities. The hybrid optimization model can generate multiple adaptive layouts instead of a single ‘optimal’ layout to satisfy different user needs by combining subjective user evaluation and intelligent optimization techniques. IGA incorporates the user’s personalized needs into the optimization process, DE enhances the global search and local optimization capabilities, and the backtracking strategy further improves the diversity and avoids local optimization. The backtracking strategy further improves the diversity and avoids local optimization. The model not only optimizes space utilization and functional fitness, but also integrates the concepts of cognitive psychology and environmental behavior to consider the impact of the layout on occupants’ comfort, stress, attention, and overall health, and to improve mental health and quality of life. In addition, the model, through a flexible user feedback mechanism, is able to adjust the layout scheme according to different cultural contexts to ensure compliance with local cultural preferences. The innovation of the study lies in the fact that it breaks through the deficiencies of traditional layout optimization methods in terms of personalized design and global optimization capabilities by combining subjective user evaluation and intelligent optimization techniques. Compared with the existing methods, the model not only enhances the global search capability, but also introduces a backtracking strategy to improve the population diversity, effectively avoids local optimization, and is able to provide more efficient and personalized indoor layout design solutions. The contribution of the study is to propose a flexible and personalized internal space layout optimization method, which combines the advantages of GA and DE, overcomes the limitations of the traditional methods. Besides, it can effectively improve the utilization of space, functional fitness, and better adapt to the user’s needs, cultural background and mental health needs, which provides an innovative solution for intelligent building layout design.

## 2. Methods and materials

Aiming at the problem of insufficient ability of diversity maintenance and global optimization in building ISL design, this study firstly constructs an initial layout generation model based on IGA. The relationship between room functions and spatial locations is characterized by a binary coding method, and a comprehensive evaluation of area ratio, functional priority and spatial connectivity is achieved by combining the dynamic fitting function. Secondly, the DE algorithm is introduced to enhance the global search and local optimization of layout schemes. The population structure is dynamically updated through mutation, crossover, and selection operations, and the backtracking strategy is combined to activate population diversity during optimization stagnation, avoiding falling into local optima. Finally, the study proposes a novel spatial layout optimization model that integrates IGA and DE. Among them, IGA denotes a GA that integrates users’ subjective evaluation into the optimization process, and adjusts the optimization direction through users’ feedback to realize personalized design. IDE denotes a DE algorithm that enhances the ability of global search and local optimization, and improves the convergence speed and optimization effect by improving the strategy.

### 2.1. Architectural interior space layout based on IGA

**Fig 1:**
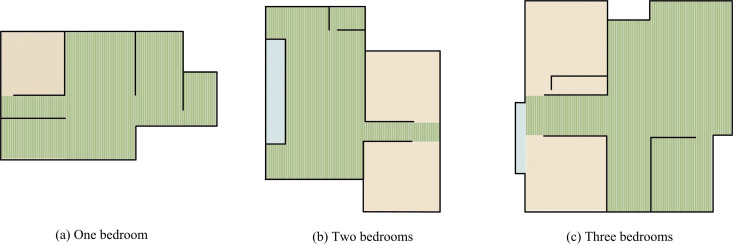
Plan frame of the house.

ISL is an important component of architectural design, which not only affects the efficiency and comfort of building use, but also directly affects the quality of life of residents. With the acceleration of urbanization, residents’ demands for functionality, comfort, and personalization of housing continue to increase [17]. However, in actual architectural design, due to the limited land resources and constraints of development costs, housing layouts gradually tend towards standardization and modularization. The common housing layouts in the current market are shown in Fig 1 [18].

**Fig 2:**
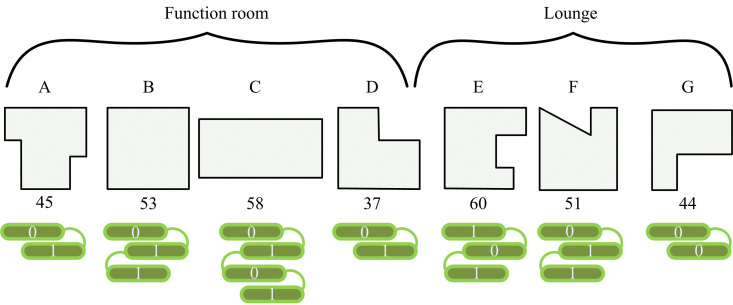
Schematic diagram of IGA chromosome coding.

Fig 1(a), 1(b), and 1(c) show the floor plan framework of a one bedroom house, a two bedroom house, and a three bedroom house, respectively. In Fig 1, most layouts focus on functional zoning and residential population needs during the design phase, such as the basic configuration of living room, bedroom, kitchen, and bathroom. However, due to the need to balance lighting, ventilation, structural safety, and development costs in unit design, it is difficult to meet the personalized needs of different family structures and living habits. This rigid spatial layout pattern often leads to residents facing problems such as low space utilization, fragmented functional areas, or unsmooth living flow in actual use [19]. Therefore, the importance of interior layout design is becoming increasingly prominent. With the rapid development of artificial intelligence technology, GA-based optimization methods have gradually become an important tool in the field of ISL design. Among them, IGA is more suitable for ISL design that meets personalized needs because it can integrate users’ subjective evaluations into the design optimization process. Through interactive design strategy, i.e., the strategy of integrating user feedback and subjective evaluation into the design optimization process, design solutions are dynamically adjusted through the interaction between the user and the system, to realize the personalized design requirements. Specifically, first, populations of initial ISLs are generated; Secondly, the fitness value is updated based on the user’s preference evaluation of candidate layout schemes; Finally, the layout scheme is iteratively optimized through genetic operations such as selection, crossover, and mutation until the final scheme that meets the design requirements is obtained [20–22]. In the encoding stage, the IGA chromosome encodes as shown in Fig 2.

In Fig 2, there are seven sub-rooms, namely A, B, C, D, E, F, and G. The corresponding arrangement serial number of each sub-room indicates its specific location relationship, e.g., room A corresponds to serial number (45), room B corresponds to serial number (53), etc. In the encoding, the character set is in the form of binary [0,1], and multiple possible layout schemes are generated through permutations and combinations. For example, if room A is located at serial number (45) and room B is located at serial number (53), the encoding of this layout can be expressed as binary encoding of 101101 for A(45) and 110101 for B(53). Through permutations and combinations, the binary encoding generates a large number of different layout scenarios, which ensures the diversity of the initial population. Each chromosome represents a specific room layout, and different codes in the population correspond to different spatial allocations and functional layouts, thus avoiding duplication and premature convergence of layout schemes. In addition, the encoding can effectively prevent the GA from falling into a local optimum at an early stage by providing a rich solution space and diverse layout schemes. The maintenance of diversity helps to increase the breadth of the search and ensures that the algorithm can explore different design schemes during the iteration process, which promotes the optimization of the convergence speed. To enhance the design rationality and adaptability of the model to architectural practice, the research team invited registered architects with more than 10 years of work experience to participate in the data labeling stage, providing professional guidance on space division, functional arrangement logic and user behavior model. The model not only analyzes and optimizes the volume of existing space, but also suggests the use of extended space according to the input conditions, such as mezzanine design and transitional space excavation. Assuming the number of rooms is N and the adjacency weight matrix between rooms is $W_{ij}$, the constraints generated by the overall initial layout are shown in equation (1).


$$L=\min\left(\sum_{i=1}^{N}\left|A_{i}-A_{avg}\right|+\sum_{i=1}^{N}\sum_{j=1}^{N}W_{ij}\cdot d_{ij}\right)$$ (1)

In equation (1), L means the objective function value of the initial layout; $A_{i}$ represents the actual area of the i th room; $A_{avg}$ represents the average area of all rooms; $W_{ij}$ represents the adjacency weight between room i and room j, with a value range of [0,1]. $W_{ij}$ is determined by a heuristic method. Specifically, the weighting values are set based on factors such as the relative location between rooms, mobility mobility and functional needs. For example, two functionally similar or connected rooms (e.g., bedroom and bathroom) may have higher weight values, while rooms that are farther apart (e.g., kitchen and living room) will have lower weights; $d_{ij}$ represents the Euclidean distance between room i and room j. In the iterative optimization stage, it is necessary to use a dynamic fitness function to rank the advantages and disadvantages of different chromosomes, especially when considering multiple attributes such as function, orientation, and space utilization. The fitness function needs to have the ability to dynamically adjust. The fitness function is defined as denoted in equation (2).


$$f_{k}=\sum_{i=1}^{N}\pi \left[\lambda_{1}\cdot \frac{S_{i}}{S_{total}}+\lambda_{2}\cdot C_{i}+\lambda_{3}\cdot F_{i}\right]$$ (2)

In equation (2), $f_{k}$ represents the k -th chromosome, which is the fitness value of the candidate layout scheme; $\lambda_{1}$, $\lambda_{2}$, and $\lambda_{3}$ represent the dynamic adjustment coefficients of area density, functional priority, and spatial connectivity, respectively. Among them, $\lambda_{1}$ weight is set according to the ratio between the importance of room area and the actual space utilization, and a larger value means that the room area has a greater influence in the overall design. $\lambda_{2}$ weight is usually adjusted according to the frequency of use of the function and the user demand. For example, the functional priority of the kitchen and the bathroom is usually higher. $\lambda_{3}$ weight reflects the design of the dynamic line in the spatial layout and the convenience of the activities of the occupants, and a higher $\lambda_{3}$ weight means that connectivity between spaces is prioritized in the layout. $S_{i}$ refers to the calculated area of the i -th room; $S_{total}$ denotes the total calculated area of the room; $C_{i}$ represents the spatial connectivity of the i -th room; $F_{i}$ denotes the functional compatibility of the i -th room. Although combining equations (1) and (2) can effectively optimize the layout scheme, genetic drift problems are prone to occur during the iteration process. Specifically, genetic drift refers to the gradual loss of population diversity over multiple iterations, ultimately leading to the convergence of the entire population to a local optimal solution, making it difficult to obtain a global optimal solution [23,24]. To this end, it is investigated that the integration of K-means clustering in the GA algorithmic framework can improve the global search capability and optimization effect by dividing the population into sub-populations, maintaining population diversity and preventing early convergence, as well as dynamically guiding the chromosome selection and crossover operations in the optimization. The objective function is shown in equation (3).


$$J=\sum_{k=1}^{K}{\sum_{i\in C_{k}}\left\|x_{i}-\mu_{k}\right\|}^{2}$$ (3)

In equation (3), J represents the clustering objective function value; K represents the number of categories in the cluster; $C_{k}$ represents the chromosome set in class k; $x_{i}$ means the eigenvector of the i -th chromosome; $\mu_{k}$ denotes the centroid vector of class k. After completing the clustering, the fitness function is re-evaluated for the chromosomes in each class, incorporating intra-class distribution information and centre-of-mass bootstrap terms. The optimized fitness function is shown in equation (4).


$$\begin{array}{c} f_{k}^{\prime }=f_{k}+\gamma \cdot \frac{\sum {\mathrm{\backslash nolimits}}_{i\in C_{k}}\left\|x_{i}-\mu_{k}\right\|}{\left|C_{k}\right|} \end{array}$$ (4)

In equation (4), $\begin{array}{c} f_{k}^{\prime } \end{array}$ denotes the optimized fitness function; γ denotes the clustering regulator; and $\left|C_{k}\right|$ means the number of chromosomes in the k -th class. The clustering results are used for the probability calculation of dynamic crossover and mutation operations, and the formula is shown in equation (5).


$$\{\begin{array}{c} {}*20cP_{c}^{\left(k\right)}=P_{c}^{\left(init\right)}\cdot \left(1-\frac{\sigma_{k}}{\sigma_{\max}}\right)\\ P_{m}^{\left(k\right)}=P_{m}^{\left(init\right)}\cdot \frac{\sigma_{k}}{\sigma_{\max}} \end{array}$$ (5)

**Fig 3:**
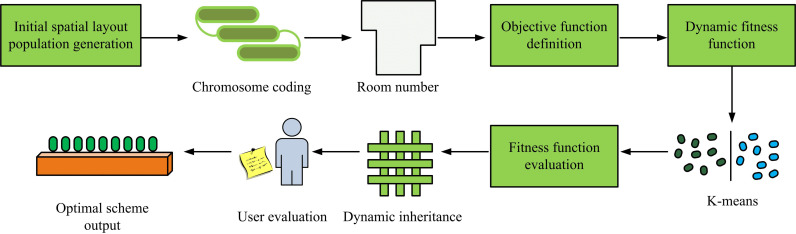
Building internal space layout process based on IGA.

In equation (5), $P_{c}^{\left(k\right)}$ and $P_{m}^{\left(k\right)}$ denote the dynamic crossover probability and dynamic variation probability of individuals in the k -th class, respectively; $P_{c}^{\left(init\right)}$ and $P_{m}^{\left(init\right)}$ denote the initial crossover and variation probabilities, respectively; $\sigma_{k}$ denotes the standard deviation of the fitness in the k -th class; $\sigma_{\max}$ denotes the maximum value of the standard deviation of the fitness in all classes. Finally, the process of building ISL based on IGA is shown in Figure 3.

As shown in Fig 3, starting from the generation of the initial population, based on the basic framework information and spatial functional requirements of the house, an initial layout scheme is constructed through binary encoding, and objective function constraints are defined to balance key factors such as room area, adjacency weight, and space utilization. Subsequently, the layout scheme is evaluated through a dynamic fitness function, and the fitness of the population is flexibly optimized by dynamically adjusting coefficients such as area proportion, functional priority, and spatial connectivity. On this basis, the K-means clustering method is introduced to divide the population into multiple sub-populations, dynamically adjust the crossover and mutation probabilities based on the fitness distribution within the class, maintain population diversity, and accelerate convergence. At the same time, by updating the fitness value based on users’ subjective evaluations, personalized needs are integrated into the optimization process, further enhancing the practicality and satisfaction of the layout. After multiple iterations, the population gradually converges to the optimal layout scheme, and finally outputs an ISL design with reasonable functionality, high space utilization, and meeting personalized needs.

### 2.2. Optimization of building internal space layout by integrating de algorithm

**Fig 4:**
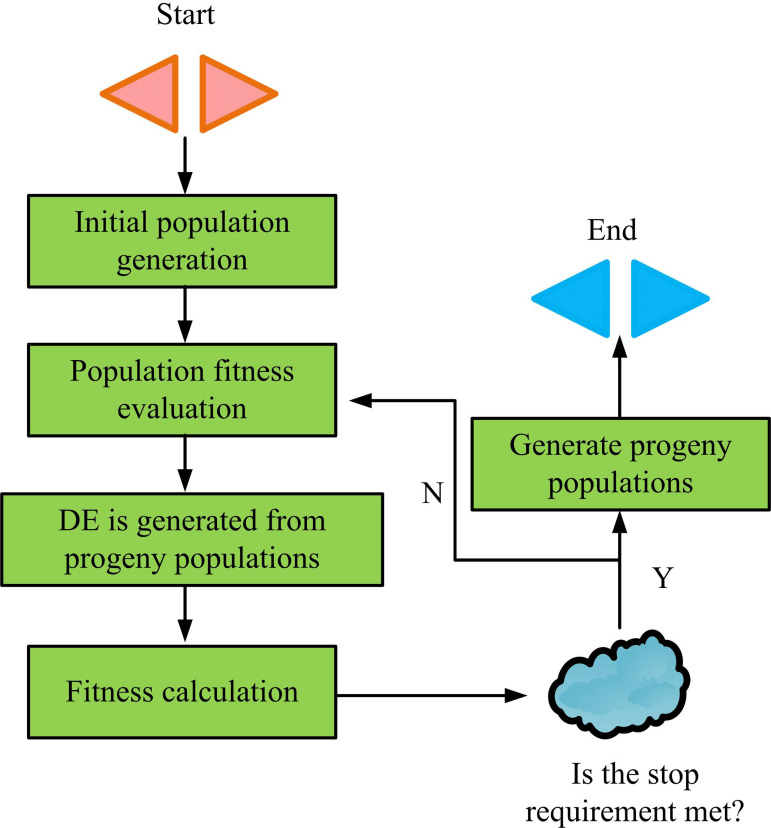
DE algorithm flow.

A method for building ISL has been developed by improving IGA. Although this interactive layout strategy can fully integrate customers’ subjective evaluations and achieve personalized design needs, its ability to maintain population diversity and global optimization is still insufficient under complex constraint conditions, and it is easy to fall into local optimal solutions [25,26]. In addition, users may experience fatigue during long-term participation in the evaluation process, which can reduce the subjectivity of the evaluation results and ultimately affect the final effect of layout optimization. To address these issues, research introduced DE to improve the optimization process of IGA. The DE algorithm, with its powerful global search capability and flexible parameter adjustment, can effectively avoid the population falling into local optima, while improving optimization efficiency and the quality of layout schemes [27,28]. The process of the DE algorithm is denoted in Fig 4.

As shown in Fig 4, the process of the DE algorithm mainly includes four key steps: population initialization, mutation operation, crossover operation, and selection operation. Firstly, in the population initialization stage, DE randomly generates several candidate solutions based on the solution space of the problem as the initial population, and evaluates the quality of the initial individuals through a fitness function. Secondly, in the mutation operation, each target individual generates a mutation vector through differential operations with other individuals, while introducing global perturbations to enhance the algorithm’s exploratory ability. Then, in the crossover operation, the mutation vector is combined with the target individual through crossover probability to generate new candidate individuals, thus achieving a balance between global search and local development [29]. Finally, in the selection operation, the candidate individuals are compared with the original individuals based on their fitness values, and the winner enters the next generation population to gradually improve the overall quality of the population. In the mutation operation, a mutation vector is generated by randomly selecting three different individuals from the population, as shown in equation (6).


$$v_{i}^{\left(g\right)}=x_{r1}^{\left(g\right)}+F\cdot \left(x_{r2}^{\left(g\right)}-x_{r3}^{\left(g\right)}\right)$$ (6)

In equation (6), $v_{i}^{\left(g\right)}$ represents the variation vector generated by the i -th individual in the g -th generation; $x_{r1}^{\left(g\right)}$, $x_{r2}^{\left(g\right)}$, and $x_{r3}^{\left(g\right)}$ represent vectors of three randomly selected individuals from the population, respectively; F denotes the mutation factor, usually between 0.5 and 1. In this case, the value 0.8 is taken to balance the exploration optimization problem. In the crossover stage, the mutation vector is combined with the current individual vector to generate the experimental vector, as shown in equation (7).


$$u_{i}^{\left(g\right)}=\{\begin{array}{c} {}*20cv_{i,j}^{\left(g\right)},ifrand(0,1)\leq CR\\ x_{i,j}^{\left(g\right)},otherwise \end{array}$$ (7)

In equation (7), $u_{i}^{\left(g\right)}$ stands for the experimental vector of the i -th individual; $v_{i,j}^{\left(g\right)}$ and $x_{i,j}^{\left(g\right)}$ respectively represent the value of the j -th dimension in the mutation vector and the value of the j -th dimension in the current individual vector. CR denotes the crossover probability, which usually ranges from 0.7 to 1. In this case, the value 0.9 is taken to improve the local optimization performance. After completion, in the selection stage, the experimental vector is compared with the target individual based on the fitness function, and the individual with better fitness is retained to enter the next generation. The formula is shown in equation (8).


$$x_{i}^{\left(g+1\right)}=\{\begin{array}{c} {}*20c\begin{array}{cc} {}*20cu_{i}^{\left(g\right)} & iff(u_{i}^{\left(g\right)}\leq f(x_{i}^{\left(g\right)})) \end{array}\\ \begin{array}{cc} {}*20cx_{i}^{\left(g\right)} & otherwise \end{array} \end{array}$$ (8)

**Fig 5:**
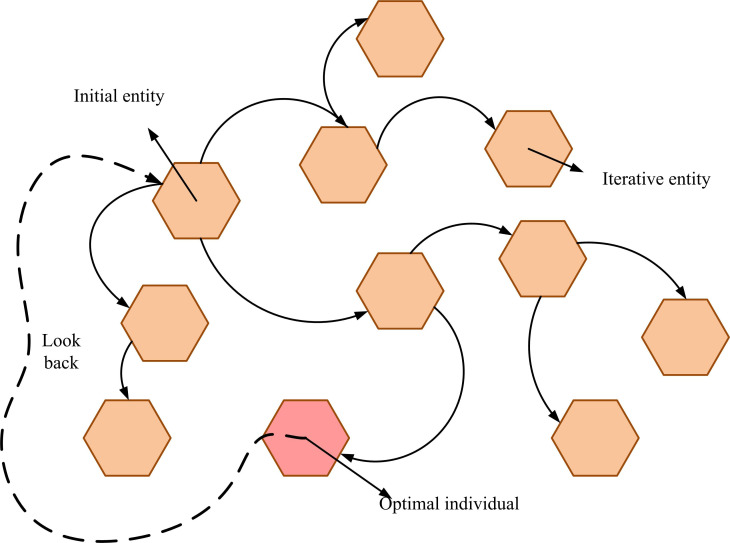
Backtracking strategy diagram.

In equation (8), $x_{i}^{\left(g+1\right)}$ denotes the vector of the i -th individual in the g+1 -th generation; f(_) denotes fitness function; $u_{i}^{\left(g\right)}$ represents the experimental vector; $x_{i}^{\left(g\right)}$ means the target individual in the current generation population. Combining equations (6), (7), and (8), the DE algorithm gradually improves the fitness level of the population through iterative operations in three stages: mutation, crossover, and selection. However, the DE algorithm still has certain limitations in complex optimization problems, such as the problem of population convergence being too fast in later iterations, leading to search stagnation at local optimal solutions. In addition, the diversity of the population gradually decreases with the increase of iteration times, weakening the global search ability of the algorithm [30]. To address this issue, an optimization mechanism based on backtracking strategy is introduced, as shown in Fig 5.

As shown in Fig 5, the backtracking strategy dynamically adjusts the search direction and range of the current population by introducing the fitness information of the historical individuals in the process of population iteration, thus effectively avoiding the problem of premature convergence of the population. Specifically, when a locally optimal solution (i.e., the red individual in the figure) stagnates during population iterations and there is no significant progress between multiple generations, the backtracking strategy improves population diversity by comparing the fitness difference between the current individual and the historically optimal individual, and reselecting and inserting the individual with higher historical fitness [31]. By using this mechanism, the global search capability is reactivated when the population falls into a local optimum, thereby improving the overall optimization performance of the algorithm. The calculation formula for searching and updating newly introduced candidate individuals is shown in equation (9).


$$x_{i}^{\left(g+1\right)}=x_{i}^{q}+\eta \cdot (h_{k}-x_{i}^{q})+\vartheta \cdot r$$ (9)

In equation (9), $x_{i}^{q}$ represents the target individual in the current population; $h_{k}$ represents the k -th individual randomly selected from the historical set of high fitness individuals; ϑ represents the disturbance factor; r represents a random vector; η represents the backtracking vector. At the same time, to achieve a balance between population diversity and optimized stability, fitness weight terms are introduced to constrain individuals undergoing backtracking adjustment, as shown in equation (10).


$$F_{opt}(x_{i}^{g+1})=F(x_{i}^{g+1})+\phi \cdot \pi \left(1-\frac{\left\|x_{i}^{g+1}-h_{k}\right\|}{\left\|h_{k}\right\|}\right)$$ (10)

**Fig 6:**
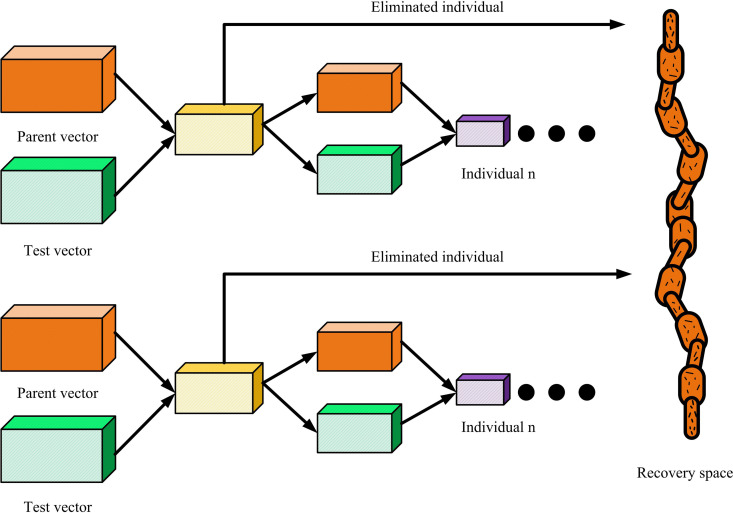
Schematic of iteration process of IDE-IIGA.

In equation (10), $F_{opt}(x_{i}^{g+1})$ represents the optimized individual fitness; ϕ represents the fitness correction coefficient; $h_{k}$ refers to individuals with high historical fitness for reference. At this point, the iterative process of improving DE combined with IGA is shown in Fig 6.

In Fig 6, this process combines the advantages of improved DE and IGA, with a focus on enhancing the algorithm’s global search capability and local optimization efficiency. Firstly, the algorithm generates an initial population based on user feedback and evaluates its fitness. Subsequently, the population is globally optimized through IDE mutation, crossover, and selection operations, while dynamically introducing historical optimal solutions and perturbation mechanisms to maintain population diversity and prevent premature convergence of the algorithm. In each iteration, based on the multi-task optimization framework, the weight factors of different tasks are adjusted in real-time to adapt to the current state of the population and the complexity of the objective function. Through multiple iterations, the algorithm continuously updates the population quality and ultimately converges to the global optimal solution. The calculation formula for dynamic weight adjustment is shown in equation (11).


$$w_{k}=\frac{\sigma_{k}^{-1}}{\sum {\mathrm{\backslash nolimits}}_{j=1}^{n}\sigma_{j}^{-1}}$$ (11)

In equation (11), $w_{k}$ represents the dynamic weight of task $T_{k}$; n means the total number of tasks; $\sigma_{k}$ represents the standard deviation of the fitness value of the population corresponding to task $T_{k}$. The calculation formula for the perturbation mechanism of the historical optimal solution is shown in equation (12).


$$x_{i}^{\left(t+1\right)}=x_{i}^{\left(t\right)}+\beta \cdot rand\cdot (x_{best}-x_{i}^{\left(t\right)})$$ (12)

**Fig 7:**
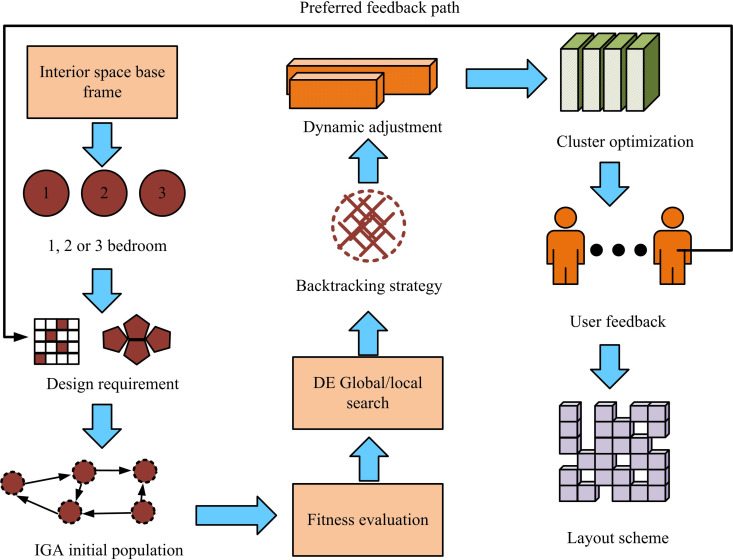
New building interior space layout model flow.

In equation (12), $x_{i}^{\left(t+1\right)}$ and $x_{i}^{\left(t\right)}$ indicate the new positions of the i -th individual in the t+1 and t generations, respectively; $x_{best}$ represents the globally optimal individual of the current population; β stands for dynamic disturbance factor. Finally, a new model for ISL in buildings is proposed, and the process of the model is denoted in Fig 7.

In Fig 7, firstly, the initial population is generated according to the basic framework of the building’s interior space and the design requirements, and the room layout information is represented by coding to ensure the diversity of the initial programs. Next, the individuals in the initial population are evaluated for adaptability, and key factors such as room area, functional partitioning, and spatial connectivity are considered comprehensively to screen out candidate solutions that meet the design requirements. During the optimization, the DE algorithm is used for global search and local optimization, and the population quality of the GA is gradually improved with the help of mutation, crossover and selection operations. When the optimization process needs to incorporate a large amount of subjective feedback from users, the advantages of IGA are more prominent, and IGA adjusts the population fitness through real-time evaluation by users to satisfy the personalized requirements more flexibly. To prevent the algorithm from falling into a local optimum, a backtracking strategy is introduced to dynamically activate the population diversity when the optimization is stalled to generate a new search direction. At the same time, the efficiency and adaptability of the algorithm is improved by dynamically adjusting the task goal weights and grouping the populations in combination with clustering optimization. A flexible, user-driven feedback loop is introduced, which directly translates users’ personalized needs into optimization directions through their subjective feedback, ensuring that the layout scheme can better match users’ actual needs and preferences. After several rounds of iteration, the population gradually converges to the final indoor space design scheme with reasonable layout, optimized functions and personalized needs. Among them, the role of user evaluation in dynamically updating the fitness function is to directly integrate users’ subjective preferences into the optimization process, and to adjust the weights in the fitness function to reflect the degree of importance that users attach to different design elements. The weights of the subjective inputs are not the same, and they are dynamically adjusted according to the user’s preference for each design factor (e.g., space utilization, functional allocation, connectivity, etc.) to ensure that the optimization results are more in line with individualized needs. The model supports two types of input modes: firstly, the importation of existing architectural drawings (e.g., CAD, BIM models) and the automatic extraction of structural and opening information; secondly, the manual drawing of spatial boundaries and the definition of the location of openings through the platform. Before running the model, the number of functional zones and expected relationships (e.g., privacy, proximity) need to be specified. Furniture arrangements are automatically matched based on an ergonomic database, or can be manually adjusted by the user. If the number of rooms specified by the user exceeds the existing spatial carrying capacity, the model will issue a warning through the constraint detection mechanism and generate the optimal compromise solution, such as merging functional zones, prompting space expansion suggestions, etc., to ensure the architectural implementability of the final result.

## 3. Results

To assess the effectiveness of the new model, a suitable experimental environment was established. Firstly, value selection tests were conducted on the two types of hyperparameters that have the greatest impact on model performance, and ablation tests were also performed on the final model. Advanced methods were compared and tested based on the number of optimal solutions, flow line optimization rate, and space utilization rate. In addition, a layout optimization comparison was conducted using real interior housing as an example, and the functional adaptability during the process was quantified to assess the practical effectiveness of the raised method. The diversity index is calculated as shown in equation (13).


$$D=-\sum_{i=1}^{n}p_{i}\log(p_{i})$$ (13)

In equation (13), D is the diversity index; n is the total number of individuals in the population; and $p_{i}$ is the proportion of i -th individuals in the population. The higher the value of this index, the greater the diversity of individuals in the population, and vice versa, the population tends to be homogeneous. The calculation of space utilization rate is shown in equation (14).


$$S=\frac{A_{used}}{A_{total}}\times 100\;\%$$ (14)

In equation (14), S denotes space utilization; $A_{used}$ denotes the area of space used; and $A_{total}$ denotes the area of total usable space.

### 3.1. Performance testing of new building internal space layout model

The study used Building Layout Dataset (BLD) and Residential Floor Plan Dataset (RFPD) as test data sources. Among them, the BLD dataset is a specialized dataset for the design and optimization of ISL in buildings, providing two-dimensional layout information for various typical houses. Each layout data included geometric information of the room, such as area, boundary length, functional labels such as living room, bedroom, kitchen, adjacency matrix, room orientation, and lighting information. RFPD contained two-dimensional floor plan data for different types of residential buildings, including information on room distribution, functional zoning, room connectivity, and space utilization. In addition, the dataset provided various constraint conditions, such as spatial lighting, structural safety, room flow, etc., which were suitable for testing the performance of complex constraint optimization models. The experimental environment and parameter settings are denoted in Table 2.

**Table 2:** Details of experimental environment and parameters.

Parameter	Configuration
CPU	Intel Core i9-12900K, base frequency 3.2 GHz
GPU	NVIDIA GeForce RTX 4090, 24 GB VRAM
Memory	64 GB DDR5
Operating system	Windows 11 Professional
Programming framework	Python 3.9 with TensorFlow 2.12.0
Optimization algorithm	Improved DE IGA
Population size	150
Max iterations	300
Learning rate	0.01
Mutation rate	0.25
Crossover rate	0.8
Selection strategy	Elitist selection
Constraint weight	1.2
Room connectivity threshold	0.6
Iteration termination criterion	10 consecutive iterations with no fitness improvement

**Fig 8:**
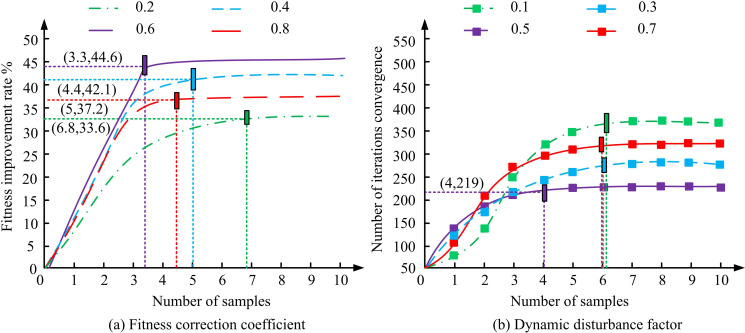
Hyperparameter selection test.

The study first normalized all input data by converting the values of each feature to zero mean and unit variance to ensure that different features have the same scale, thus avoiding the excessive influence of certain features on model training. Next, the dataset was divided according to the different types and complexities of room layouts in an 80%/20% ratio, where 80% was used as the training set, which contains a variety of room combinations and diversity of functional requirements to ensure that the model is able to learn a wide range of layout patterns. The remaining 20% was used as the test set to evaluate the generalization ability of the model. Based on the parameter configuration in Table 2, the study first conducted value selection tests on the two types of hyperparameters that have the greatest impact on the model, namely the fitness correction coefficient ϕ and the dynamic disturbance factor β. The test results are shown in Fig 8.

**Fig 9:**
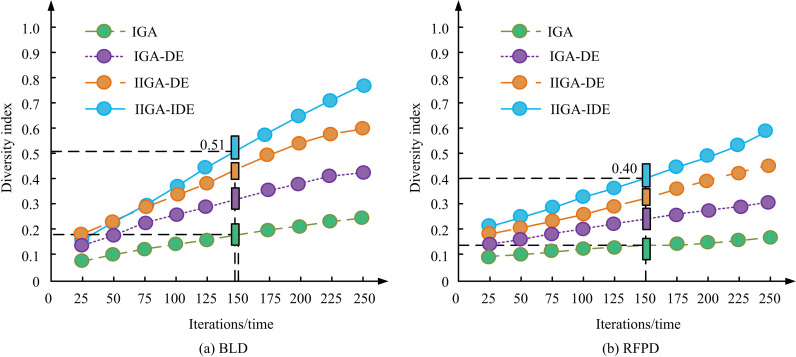
Ablation test results.

Fig 8 (a) showcases the results of the fitness correction coefficient selection test, and Fig 8 (b) showcases the results of the dynamic disturbance factor selection test. The data points in the figure indicate the performance improvement rate and the number of iterations required for convergence obtained by adjusting the values of the fitness correction factor and the dynamic interference factor at different sample sizes. In Fig 8(a), the performance improvement rate reached a maximum value of 44.6% when the correction factor was 0.6, while the performance improvement decreased significantly to only about 42.1% when the correction factor was lower than 0.4. This indicated that the fitness correction factor had a large impact on the global optimization capability. To further explore the sensitivity of this parameter, higher correction coefficients (e.g., 0.8) may lead to premature bias of the population towards the historical optimal solution, thus limiting the diversity of the search. Similarly, Fig 8(b) shows that the model reached convergence at the 219th iteration when the perturbation factor was 0.5, whereas smaller perturbation factors (e.g., 0.1) required more iterations (about 350) to converge. This indicated that the perturbation factor had a significant effect on the convergence speed, and too high a perturbation factor may lead to a decrease in the stability of the population, which affects the stability of the optimization process. The results also illustrated that the choice of hyperparameters had a significant sensitivity to the optimization process. Therefore, the study finally selected a correction factor of 0.6 and a dynamic perturbation factor of 0.5 as the optimal hyperparameter configuration of the model, and the results of this selection were based on experimental tuning, which is determined after several rounds of parameter adjustment. Specifically, too large or too small selection of correction coefficient and dynamic perturbation factor would make the iteration speed and global optimization ability of the model decrease. The study continued with ablation testing, and the test findings are indicated in Fig 9.

Fig 9(a) and 9(b) show the ablation test results in the BLD dataset and RFPD dataset. From Fig 9(a), the diversity indices of the four models gradually increased with the increase in the number of iterations. The diversity index of the IIGA-IDE model reached 0.51 at the 125th iteration, which is significantly higher than that of the other three models, demonstrating its superiority in maintaining the diversity of the population and enhancing the global search capability. In contrast, traditional ISL design methods are usually difficult to maintain high diversity in more complex layouts, resulting in less efficient optimization. In Fig 9(b), the IIGA-IDE model showed a clear diversity advantage after 100 iterations, and its diversity index reached 0.40 at the 150th iteration, which was much higher than that of the IIGA-DE model (0.35) and the IGA-DE model (0.30). Results suggested that the IIGA-IDE was able to adapt to the complex constraints more efficiently and showed stronger robustness in the optimization problems with higher diversity requirements and exhibited strong robustness and performance advantages. Compared with the benchmark results, the optimization efficiency and result quality of IIGA-IDE were better than those of the traditional methods, which further validated the powerful capability of the model in complex indoor space layout. The study introduced meta-heuristics, such as PSO and ABC, as well as more advanced algorithmic models for comparison. Examples included Deep Reinforcement Learning Optimization (DRLO), Graph Neural Network Optimization (GNNO), Multi-Objective Evolutionary Design Network (MOED), and Evolutionary Design Network (MOED-Net). These approaches are competing models that have excelled in optimization problems in recent years. DRLO optimizes the decision-making process through a reward mechanism and is suitable for dynamic and complex environments; GNNO models the node relationships in an optimization problem through a graph structure, which is advantageous when dealing with spatial layout problems; MOED-Net combines a multi-objective optimization and an evolutionary strategy to efficiently deal with optimization of multiple design objectives. These models were chosen because they show better performance in global optimization, learning capability and multi-objective design, which can be used as comparison models to effectively evaluate the advantages of IIGA-IDE. The test outcomes are denoted in Table 3.

**Table 3:** Test results of different algorithms.

Date set	Model	Diversity index	Moving line optimization rate/%	Space utilization rate	Calculate the cost/s	Convergence rate (times per second)	Layout feasibility/%	User experience satisfaction/%	*p*
BLD	DRLO	28	82.34	0.85	12.54	113.45	85.69	81.25	0.038
	GNNO	30	85.47	0.88	10.32	94.25	88.75	84.62	0.029
	MOED-Net	32	87.21	0.91	9.76	102.28	90.34	88.45	0.021
	PSO	31	88.91	0.84	11.37	99.83	‌92.15‌	‌89.80‌	‌0.017‌
	ABC	33	89.29	0.86	9.25	127.3	‌91.40‌	‌88.90‌	‌0.024‌
	IIGA-IDE	36	92.83	0.96	8.14	153.4	‌96.50‌	‌98.30‌	‌0.001‌
RFPD	DRLO	26	81.67	0.83	13.23	89.26	83.24	79.81	0.047
	GNNO	29	84.98	0.87	11.45	97.14	87.12	83.45	0.033
	MOED-Net	33	89.12	0.92	9.88	99.52	85.96	91.25	0.018
	PSO	29	85.68	0.90	10.44	102.73	89.77	89.74	0.004
	ABC	27	87.71	0.91	11.28	111.29	90.15	92.54	0.003
	IIGA-IDE	38	94.76	0.97	8.06	148.79	‌95.60‌	‌97.80‌	‌0.002

According to Table 3, the IIGA-IDE algorithm performed the best in all test metrics. Both on the BLD dataset and the RFPD dataset, its number of optimal solutions reached 36 and 38, respectively, which was significantly higher than the other comparison algorithms. Its kinematic optimization rates were 92.83% and 94.76%, which were more than 5% higher than the MOED-Net algorithm, and also significantly higher than the traditional methods GNNO and DRLO. This performance improvement is mainly attributed to the flexible adjustment mechanism of IIGA-IDE in spatial layout optimization, which can effectively adjust the layout according to different spatial demands and avoid falling into local optimums through the backtracking strategy. The space utilization rates both reached 0.96 and 0.97, which were significantly better than the traditional methods such as GNNO and DRLO, indicating that IIGA-IDE has excellent optimization ability in spatial layout design. Compared with other algorithms, IIGA-IDE was able to better balance the functional requirements of each room with the efficiency of space utilization, thus improving the overall space design quality. In addition, the average computational time cost of IIGA-IDE was 8.14 seconds and 8.06 seconds, respectively, which was more than 5 seconds less than that of the DRLO algorithm, which was the least computationally efficient algorithm, showing its advantage in computational efficiency. In terms of convergence speed, IIGA-IDE had a convergence rate of 153.4 times/second on the BLD dataset and 148.79 times/second on the RFPD dataset, which was significantly higher than other algorithms, reflecting its advantage in fast convergence. In terms of user experience satisfaction, IIGA-IDE’s satisfaction on the BLD dataset and RFPD dataset was 98.30% and 97.80% respectively, which was significantly higher than that of algorithms such as PSO and ABC, demonstrating its superiority in meeting the user’s individualized needs and optimizing the practical effects of design. *p*-values were all less than 0.05, which further proved that the IIGA-IDE was statistically significant in the various tests. In summary, IIGA-IDE shows significant advantages in terms of motion optimization, space utilization enhancement, computational cost, convergence speed, and user experience satisfaction, and is the most efficient and reliable solution for the current complex layout optimization task.

### 3.2. Simulation testing of new internal space layout model for buildings

**Fig 10:**
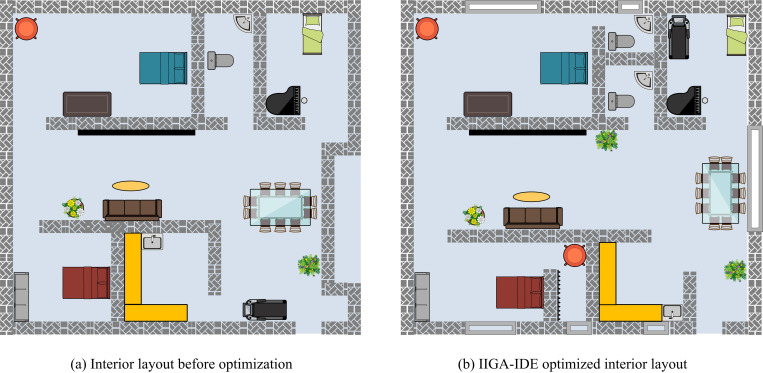
Comparison results before and after internal layout optimization.

The residential floor plan data used in the experiment were obtained from the public planning approval information and typical commercial house type samples of a high-density residential area in Nanshan District, Shenzhen. The research team collected and screened the data from March to May 2023 through the planning data platform of Shenzhen Municipal Housing and Construction Bureau, the public listing information of real estate agents and the public display projects of architectural design institutes. A total of 32 sets of 90 ~ 140 square meters three-bedroom residential floor plans were collected, covering a variety of layout types such as horizontal halls, vertical halls, suite-type and compact-type. The data selection criteria include: ① clear structure of the house type with typical functional areas such as master bedroom, second bedroom, living room, kitchen, etc.; ② real door and window openings, location of load-bearing walls and furniture arrangement information; ③ structural features applicable to medium and high-rise residential buildings. Some of the data are simplified into planar geometrical models by manual abstraction so that they can be input into the optimization model for structural processing. The study did not involve any commercial project data with OCT Shenzhen or the author’s organization, nor did it use internal data sources; all experimental data were obtained based on open channels, in compliance with the code of ethics and data use. To assess the practical application effect of the new ISL model of the building, a study was conducted on the renovation and optimization of the interior space of a three bedroom and one living room as an example. The optimization of the three houses was carried out based on the principles of functionality, ventilation and lighting, richness, and livability. The internal layout before and after optimization was compared, and the results are indicated in Fig 10.

**Fig 11:**
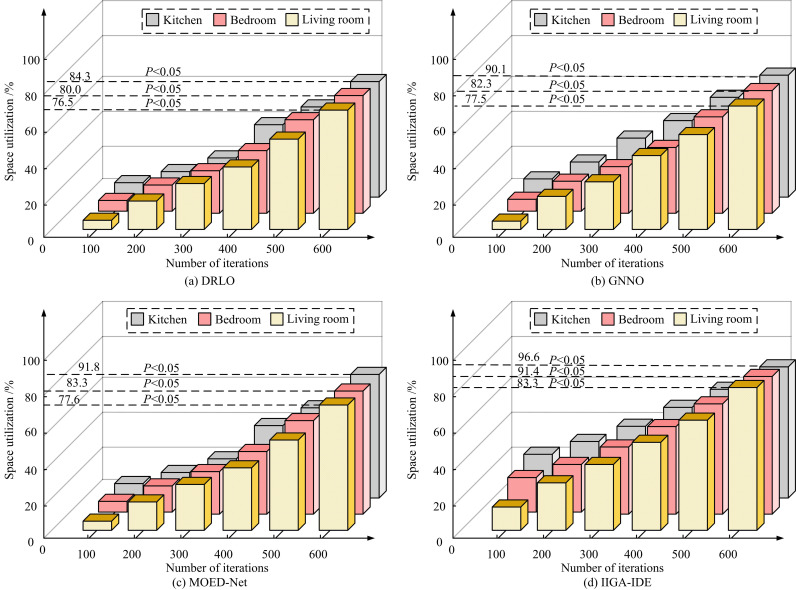
Test results of space utilization in different rooms.

Fig 10(a) and 10(b) show the interior layout before optimization and after optimization. From Fig 10, in the pre-optimized interior layout, the functional zoning of the rooms was relatively simple and the flow design was not reasonable enough, such as the distance between the bedroom and bathroom being too far, which makes daily use not convenient enough. The poor connectivity between the kitchen and dining room increased inconvenience during use. In addition, the placement of some furniture led to low space utilization, especially in the living room area which appears crowded. By dynamically adjusting the functional zoning and dynamic layout of the rooms, the IIGA-IDE model not only shortened the dynamic distances between the main functional areas, for example, the distance between the bedroom and the bathroom has been reduced by 18%, and the connectivity between the kitchen and the dining room has been improved by 20%. Space utilization has also been significantly improved, with the optimized layout increasing space utilization in the living room area by 10% and overall space utilization by 12%. The rearrangement of furniture has made the living room more open, and the overall ventilation and lighting effect has been significantly improved, and the richness and livability of the space has been further enhanced. The study tested the spatial utilization of four models in the kitchen, bedroom, and living room, and the test results are shown in Fig 11.

**Fig 12:**
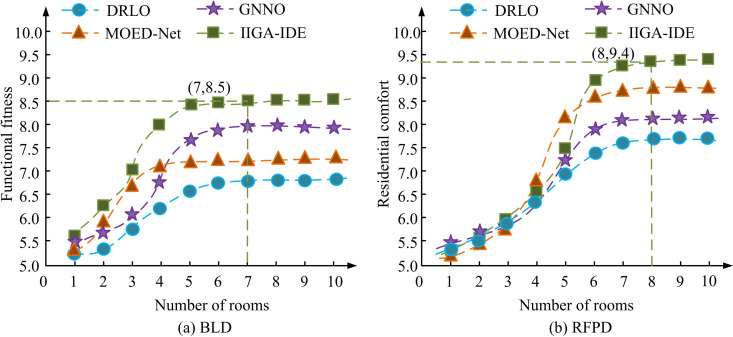
Functional fitness test results of different algorithms.

Fig 11(a), (b), (c), and (d) show the utilization test results of DRLO, GNNO, MOED, and IIGA-IDE for three types of interior spaces, respectively. In Fig 11, the IIGA-IDE model performed optimally in all tested indoor spaces, with 83.3% (*p* < 0.05), 91.4% (*p* < 0.05), and 96.6% (*p* < 0.05) space utilization in the kitchen, the bedroom, and the living room, respectively, and had the fastest convergence rate, which could be stabilized after 400 iterations (*p* < 0.05). Compared with other models, IIGA-IDE had a particularly significant improvement in space utilization in the living room, which was about 4.8% higher than MOED-Net (*p* < 0.05). Although MOED-Net had a better overall performance than GNNO and DRLO, it is still slightly inferior to IIGA-IDE in terms of iterative efficiency (*p* < 0.05).GNNO and DRLO had slower convergence speeds, and the final space utilization was relatively low (*p* < 0.05), which makes it difficult to satisfy the demand for efficient optimization. The results of the functional fitness test also showed that IIGA-IDE significantly outperformed the other models in a number of optimization metrics, with *p*-values less than 0.05, indicating that the results were statistically significant. Among them, functional adaptability refers to the flexibility and adaptability of spatial layout in meeting different functional needs and use conditions, ensuring that the layout can be effectively adjusted according to different environments and user needs. The outcomes are denoted in Fig 12.

Fig 12(a) shows the functional fitness results of different algorithms in the BLD dataset, and Fig 12(b) shows the occupant comfort results of different algorithms in the RFPD dataset. As shown in Fig 12 (a), IIGA-IDE exhibited the highest functional adaptability on both datasets. In the BLD dataset, when the number of rooms reached 7, the functional adaptability of IIGA-IDE reached 8.5, significantly better than MOED-Net’s 8.1, GNNO’s 7.6, and DRLO’s 6.9. In the RFPD dataset, IIGA-IDE achieved a living comfort level of 9.0 at a room number of 7, which was significantly ahead of MOED-Net’s 8.7, GNNO’s 7.8, and DRLO’s 7.2. By optimizing the spatial layout, IIGA-IDE not only improved the space utilization rate, but also effectively enhanced the user’s living comfort, especially in the areas of room functional zoning, dynamic design, and spatial connectivity. Compared with traditional algorithms, IIGA-IDE better considered the actual needs of users, such as the reasonable configuration of the distance between the bedroom and the bathroom, the connectivity between the kitchen and the dining room, etc., and the optimized spatial layout is more in line with the comfort of daily use. It can be seen that with the increase of the number of rooms, the proposed method can effectively expand its optimization capability, and by adding more rooms and functional constraints, IIGA-IDE can maintain high space utilization and optimization efficiency. To verify the model’s adaptability in the actual architectural design process, the study invited three senior interior designers to anonymously evaluate 10 groups of randomly generated spatial layout scenarios. The scoring dimensions included: average processing time, number of model parameters, and user satisfaction. The designers believe that the model has certain reference value in standard scenarios, but still requires manual intervention by architects in the expression of personalized needs and the shaping of spatial atmosphere. In addition, the result visualization diagram was clearly labeled with each door and window opening, furniture configuration and pedestrian path, which helps architectural professionals to quickly understand the system deduction logic and spatial organization structure. The results are shown in Table 4.

**Table 4:** Multiple index test results of different methods.

Environment	Model	Average processing time (s)	Parameters (Million)	User satisfaction (%)	*p*
Kitchen	DRLO	12.54	4.36	81.47	0.017
	GNNO	10.92	3.78	85.23	0.024
	MOED-Net	9.67	3.45	88.36	0.008
	IIGA-IDE	7.34	2.89	93.87	0.006
Bedroom	DRLO	13.76	4.67	79.52	0.008
	GNNO	11.53	3.92	83.75	0.018
	MOED-Net	9.89	3.61	86.84	0.023
	IIGA-IDE	7.68	2.94	92.43	0.002
Living room	DRLO	14.23	4.72	80.18	0.034
	GNNO	11.87	3.85	84.61	0.021
	MOED-Net	10.12	3.57	87.92	0.009
	IIGA-IDE	7.82	2.91	94.21	0.015

According to Table 4, IIGA-IDE performed superiorly in the three key metrics of average processing time, number of model parameters, and user satisfaction. In the kitchen environment, its processing time was 7.34 seconds, which was 24.1% shorter than MOED-Net, and the number of parameters was only 2.89M, which was significantly smaller than DRLO’s 4.36M, and the user satisfaction reached 93.87%, which was 12.4% higher than that of DRLO. In the bedroom environment, the processing time was 7.68 seconds, which was 44.2% less than that of DRLO, and the satisfaction rate was 92.43%, which was higher than MOED-Net’s 86.84%. In the living room environment, the model also achieved optimal performance, with a processing time of 7.82 seconds, a parameter count of 2.91M, and a satisfaction level of 94.21%, which was statistically significant (*p* = 0.015). In summary, IIGA-IDE demonstrates the advantages of low computational overhead, lightweight model and high user acceptance in different functional spaces, which verifies its practicality and reliability in layout optimization tasks. Considering its excellent performance, the method has a strong potential for application and promotion, and can be used in intelligent design of residential spaces, interior home decoration assistance systems, and real estate display and interaction platforms, providing technical support for the digital transformation of related industries.

## 4. Conclusion

A new optimization model that integrates IGA and DE algorithm was proposed to address the shortcomings of traditional architectural ISL design methods in diversity maintenance and global optimization capabilities. The experiment findings denoted that when the correction coefficient was 0.6 and the dynamic disturbance factor was 0.5, the maximum performance improvement rate of the model was 44.6%. Compared to individual IGA or DE modules, the final IIGA-IDE effectively maintained population diversity and enhanced global search capabilities by combining the advantages of improved IGA and DE algorithms, with a maximum diversity index of 0.51. Compared with other advanced algorithms, the algorithm proposed by the research had a maximum of 38 optimal solutions, the highest dynamic optimization rate of 94.76%, the highest space utilization rate of 0.97, and the shortest average processing time of 8.06 seconds. After optimizing the layout of an interior space with three bedrooms and one living room, it was found that the proposed method not only dynamically adjusted the physical space, but also effectively improved comfort, lighting, richness, and functionality. The new model had the highest space utilization rates of 96.6%, 91.4% and 83.3% for the kitchen, bedroom and living room respectively. The highest functional adaptability was 9.4, the lowest parameter quantity was 2.89M, and the highest user satisfaction was close to 94.21%. In summary, although the proposed model can effectively improve the layout effect of existing interior spaces, its optimization ability in high-dimensional spaces and more complex multi constraint scenarios still needs to be improved. In the future, further exploration can be conducted to combine deep reinforcement learning with multi-objective evolutionary algorithms to enhance the scalability and adaptability of the algorithm, while incorporating new technologies such as virtual reality to optimize user interaction experience.

Although the method performs well in optimizing room layouts, it still faces many challenges in practical applications. First, for irregular room shapes and interference from environmental factors, traditional spatial layout optimization methods usually assume that the rooms are regular rectangles or squares and fail to adequately consider the combined impact of these factors on the layout design. Therefore, future models need to be further optimized to accommodate rooms of different shapes and to effectively handle the challenges posed by geometric irregularities. At the same time, dynamic assessment of environmental factors is introduced to include external variables such as light and noise as part of the optimization goal, so as to optimize the spatial layout while enhancing the comfort and psychological experience of the occupants.

Second, with the increase in data volume, the model may face a significant increase in computational complexity and memory consumption, leading to a decrease in optimization efficiency. In addition, the sensitivity of IIGA-IDE to initial conditions is a potential problem. Different initial layouts may cause the algorithm to get trapped in local optimal solutions, especially in high-dimensional spaces and multiple constraints, and the model may need more strategies to enhance the global search capability to avoid early convergence.

Third, with the development of AI-driven layout optimization, the issue of cultural adaptation also requires urgent attention. Currently, the proposed method assumes a universal optimization criterion, but in the context of globalization and cultural diversity, the layout design should be able to adapt locally to different cultural norms. For example, the need for privacy in Eastern cultures differs significantly from the preference for open spaces in Western cultures. Future work should consider how to make layout optimization methods culturally adaptable to meet spatial needs in different cultural contexts.

Fourth, the AI space optimization system proposed in this study has outstanding performance in improving design efficiency and saving the time for exploring solutions in the early stage, but the results still need to be reviewed and adjusted by designers with a sense of architectural aesthetics, cultural understanding and humanistic care. The current model can be used as an “intelligent auxiliary tool” for architects to batch generate initial plans and explore spatial possibilities, but it is not enough to replace the role of architects in the spirit of the place, the use of scenarios, cultural adaptation and other in-depth judgments.

Finally, future work should consider how layout optimization methods can be made culturally adaptable to meet spatial needs in different cultural contexts. The concept of internal space encompasses not only mathematical efficiency, but also intersects with the domains of human emotion, cognition, and personal identity. In this era of increasing cultural diversity and personalized needs, it is crucial to meet the diverse needs of human beings through intelligent optimization design. Meanwhile, future research should explore how to bridge the gap between computational efficiency and human-centered design principles to provide more personalized and psychologically responsive spatial layouts. In addition, future research should further enhance the human-computer synergy mechanism so that AI can more effectively serve architectural practice while respecting architectural logic and user experience.

## Supporting information

S1 File Minimal Data Set Definition. (DOC)
