# Supplementary material for: Optimization design of internal space layout of three-bedroom residential apartment based on IGA and DE algorithm
Source: PLoS One. 2025 Jul 7;20(7):e0326153. doi: 10.1371/journal.pone.0326153 (PMC12233238; doi:10.1371/journal.pone.0326153)
Supplement: S1 File — (DOC) [file pone.0326153.s001.doc]

**Figure 8. Hyperparameter selection test**

| Sample Size | Fitness Improvement Rate (Correction=0.2) | Fitness Improvement Rate (Correction=0.4) | Fitness Improvement Rate (Correction=0.6) | Fitness Improvement Rate (Correction=0.8) | Convergence Iterations (Disturbance=0.1) | Convergence Iterations (Disturbance=0.3) | Convergence Iterations (Disturbance=0.5) | Convergence Iterations (Disturbance=0.7) |
| --- | --- | --- | --- | --- | --- | --- | --- | --- |
| 1 | 18.3 | 20.2 | 22.1 | 21.7 | 152 | 128 | 104 | 95 |
| 2 | 25.7 | 29.4 | 31.8 | 30.1 | 204 | 174 | 149 | 136 |
| 3 | 29.6 | 34.1 | 36.4 | 34.9 | 255 | 215 | 187 | 173 |
| 4 | 31.8 | 36.3 | 39.5 | 37.4 | 289 | 241 | 207 | 192 |
| 5 | 33.2 | 38.2 | 41.3 | 38.9 | 310 | 261 | 219 | 203 |
| 6 | 34.7 | 39.4 | 42.7 | 39.7 | 326 | 273 | 225 | 210 |
| 7 | 34.3 | 40.5 | 43.5 | 40.2 | 337 | 281 | 229 | 215 |
| 8 | 34.5 | 41.2 | 44.5 | 40.9 | 345 | 288 | 233 | 218 |
| 9 | 34.6 | 41.7 | 44.4 | 41.9 | 349 | 292 | 236 | 221 |
| 10 | 34.6 | 42.1 | 44.6 | 42.4 | 350 | 295 | 238 | 222 |

**Figure 9. Ablation test results**

| Iterations | IGA_BLD | IGA-DE_BLD | IIGA-DE_BLD | IIGA-IDE_BLD | IGA_RFPD | IGA-DE_RFPD | IIGA-DE_RFPD | IIGA-IDE_RFPD |
| --- | --- | --- | --- | --- | --- | --- | --- | --- |
| 25 | 0.17 | 0.22 | 0.28 | 0.33 | 0.15 | 0.29 | 0.24 | 0.29 |
| 50 | 0.22 | 0.26 | 0.31 | 0.37 | 0.18 | 0.22 | 0.27 | 0.31 |
| 75 | 0.23 | 0.29 | 0.35 | 0.41 | 0.26 | 0.25 | 0.33 | 0.33 |
| 100 | 0.26 | 0.32 | 0.38 | 0.44 | 0.22 | 0.27 | 0.32 | 0.36 |
| 125 | 0.29 | 0.34 | 0.41 | 0.47 | 0.24 | 0.35 | 0.34 | 0.38 |
| 150 | 0.31 | 0.36 | 0.43 | 0.49 | 0.26 | 0.32 | 0.35 | 0.47 |
| 175 | 0.33 | 0.39 | 0.46 | 0.52 | 0.28 | 0.34 | 0.37 | 0.42 |
| 200 | 0.36 | 0.42 | 0.49 | 0.55 | 0.36 | 0.36 | 0.39 | 0.44 |
| 225 | 0.38 | 0.45 | 0.52 | 0.58 | 0.32 | 0.38 | 0.41 | 0.46 |
| 250 | 0.41 | 0.47 | 0.55 | 0.61 | 0.34 | 0.39 | 0.43 | 0.48 |

**Figure 11. Test results of space utilization in different rooms**

| Model | Room | Itr-100 | Itr-200 | Itr-300 | Itr-400 | Itr-500 | Itr-600 |
| --- | --- | --- | --- | --- | --- | --- | --- |
| DRLO | Kitchen | 13.5 | 25.7 | 37.4 | 49.2 | 61.2 | 76.5 |
| DRLO | Bedroom | 16.8 | 30.1 | 42.9 | 55.4 | 68.1 | 84.3 |
| DRLO | Living Room | 17.6 | 32.8 | 45.9 | 58.7 | 71.5 | 90.1 |
| GNNO | Kitchen | 14.2 | 27.3 | 39.5 | 51.4 | 63.2 | 77.5 |
| GNNO | Bedroom | 17.2 | 32.3 | 44.3 | 56.8 | 69.1 | 82.3 |
| GNNO | Living Room | 18.1 | 34.4 | 47.7 | 59.5 | 72.1 | 90.1 |
| MOED-Net | Kitchen | 14.7 | 28.9 | 41.2 | 53.6 | 66.1 | 83.3 |
| MOED-Net | Bedroom | 18.4 | 33.8 | 47.6 | 60.1 | 72.5 | 91.8 |
| MOED-Net | Living Room | 19.2 | 35.7 | 48.9 | 62.4 | 74.4 | 93.5 |
| IIGA-IDE | Kitchen | 15.6 | 30.1 | 43.6 | 57.2 | 70.4 | 83.3 |
| IIGA-IDE | Bedroom | 20.1 | 35.7 | 50.3 | 65.5 | 78.2 | 91.4 |
| IIGA-IDE | Living Room | 21.4 | 38.9 | 53.1 | 67.2 | 80.1 | 96.6 |

**Figure 12. Functional fitness test results of different algorithms**

| Number of Rooms | DRLO (BLD) | MOED-Net (BLD) | GNNO (BLD) | IIGA-IDE (BLD) | DRLO (RFPD) | MOED-Net (RFPD) | GNNO (RFPD) | IIGA-IDE (RFPD) |
| --- | --- | --- | --- | --- | --- | --- | --- | --- |
| 1 | 5.4 | 5.6 | 5.7 | 5.8 | 5.5 | 5.7 | 5.8 | 5.9 |
| 2 | 5.6 | 5.9 | 6.1 | 6.3 | 5.8 | 6.9 | 6.1 | 6.2 |
| 3 | 5.9 | 6.3 | 6.6 | 6.9 | 6.8 | 6.3 | 6.6 | 6.8 |
| 4 | 6.3 | 6.9 | 7.4 | 7.4 | 6.5 | 6.8 | 7.6 | 7.4 |
| 5 | 6.8 | 7.4 | 7.5 | 8.1 | 7.7 | 7.3 | 7.6 | 8.2 |
| 6 | 7.2 | 7.7 | 7.9 | 8.4 | 7.5 | 8.2 | 8.4 | 8.7 |
| 7 | 7.3 | 8.1 | 8.1 | 8.5 | 7.9 | 8.4 | 8.3 | 8.9 |
| 8 | 7.5 | 8.1 | 8.2 | 8.5 | 8.1 | 8.7 | 8.4 | 9.1 |
| 9 | 7.6 | 8.1 | 8.2 | 8.5 | 8.2 | 8.7 | 8.4 | 9.2 |
| 10 | 7.6 | 8.1 | 8.2 | 8.5 | 8.2 | 8.7 | 8.4 | 9.3 |
